# Assessment of tegumental damage to *Schistosoma mansoni* and *S. haematobium* after *in vitro* exposure to ferrocenyl, ruthenocenyl and benzyl derivatives of oxamniquine using scanning electron microscopy

**DOI:** 10.1186/s13071-018-3132-x

**Published:** 2018-11-06

**Authors:** Valentin Buchter, Jeannine Hess, Gilles Gasser, Jennifer Keiser

**Affiliations:** 1 Department of Medical Parasitology and Infection Biology, Swiss Tropical and Public Health institute, Socinstrasse 57, 4051 Basel, CH Switzerland; 2 University of Basel, P.O. Box, 4003 Basel, CH Switzerland; 3 Department of Chemistry, University of Zurich, Winterthurerstrasse 190, 8057 Zurich, CH Switzerland; 4 Chimie ParisTech, PSL University, Laboratory of Inorganic Chemical Biology, F-75005 Paris, France

**Keywords:** Schistosomiasis, Organometallic derivatives, Oxamniquine, *Schistosoma mansoni*, *Schistosoma haematobium*, Scanning electron microscopy

## Abstract

**Background:**

Schistosomiasis is one of the most harmful parasitic diseases worldwide, praziquantel being the only drug in widespread use to treat it. We recently demonstrated that ferrocenyl, ruthenocenyl and benzyl derivatives of oxamniquine (Fc-OXA, Rc-OXA and Bn-OXA) are promising antischistosomal drug candidates.

**Methods:**

In this study we assessed the tegumental damage of these three derivatives of oxamniquine using scanning electron microscopy. Adult *Schistosoma mansoni* and *S. haematobium* were exposed to a concentration of 100 μM of each drug and incubated for 4–120 h, according to their onset of action and activity.

**Results:**

While on *S. mansoni* the fastest acting compound was Fc-OXA, which revealed high activity after 4 h of incubation, on *S. haematobium*, Rc-OXA revealed the quickest onset, being lethal on all males within 24 h. In both species studied, the three derivatives showed the same patterns of tegumental damage consisting of blebs, sloughing and tegument rupturing all over the body. Additionally, on *S. mansoni* distinct patterns of tegumental damage were observed for each of the compounds: tissue ruptures in the gynaecophoric canal for Fc-OXA, loss of spines for Rc-OXA and oral sucker rupture for Bn-OXA.

**Conclusions:**

Our study confirmed that Fc-OXA, Rc-OXA and Bn-OXA are promising broad spectrum antischistosomal drug candidates. All derivatives show fast *in vitro* activity against *S. mansoni* and *S. haematobium* while validating the previous finding that the parent drug oxamniquine is less active *in vitro* under the conditions described. This work sets the base for further studies on the identification of a lead oxamniquine derivative, with the aim of identifying a molecule with the potential to become a new drug for human use.

## Background

**Fig. 1:**
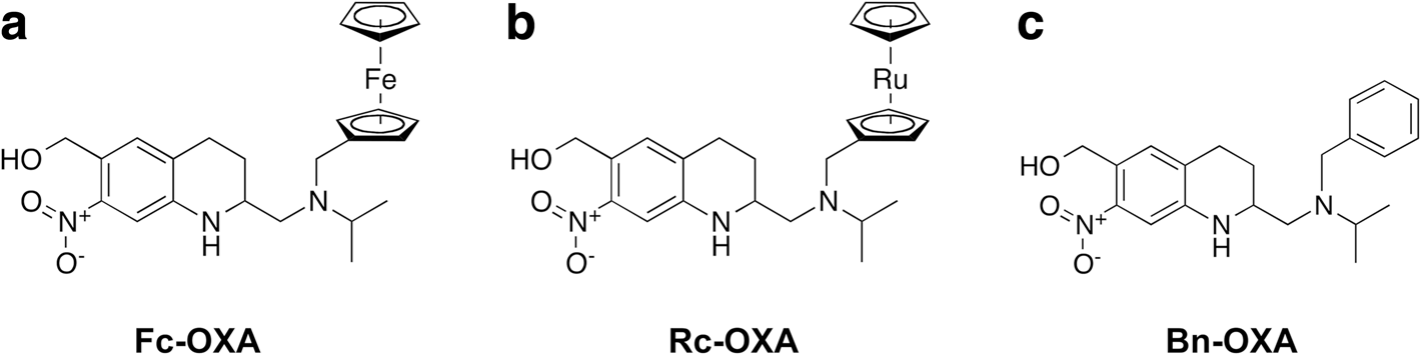
Molecular structure of the derivatives of oxamniquine. **a** Ferrocenyl oxamniquine. **b** Ruthenocenyl oxamniquine. **c** Benzyl oxamniquine

Schistosomiasis is a human parasitic disease that affected 230 million people worldwide in 2014 [1] and is caused by the infection with one or more of the six *Schistosoma* species: *Schistosoma mansoni*, *S. haematobium*, *S. japonicum*, *S. mekongi*, *S. intercalatum* and *S. guineensis*; the first three are the most important human species [1]. Because no vaccine is available, and the use of molluscicides to control the intermediate host is difficult to conduct, the most commonly used strategy to control the disease is preventative chemotherapy through large-scale administration of praziquantel [2]. Praziquantel, developed in the 1970’s, is safe and effective, but has some shortcomings: the tablets are big because of the large dose needed, it is very bitter [3] and the drug is not active against juvenile forms of the parasite [4]. Frequent retreatment of patients is therefore required to target all parasite stages. Oxamniquine (OXA) is a drug that was developed in the 1960’s [5] and proved to be effective and safe, but has two shortcomings: it is only active against *S. mansoni* [5–7] and resistance developed quickly due to punctual mutations to the enzyme’s active site [8]. OXA is a pro-drug that for activation needs to be taken up by the worm and sulfonated by a sulfotransferase (SmSULT) to an unstable intermediate that spontaneously decays to a highly electrophilic molecule that alkylates DNA, proteins and macromolecules, thus interfering with its metabolic functions and killing the parasite [9]. Enzyme orthologs are present also in *S. japonicum* and *S. haematobium* and in mammalian cells, but differences in the interaction in the active site prevent the activation of the molecule (70% protein sequence homology between *S. haematobium* and *S. mansoni*) [9]; therefore the drug is not active against *S. haematobium* [6]. The SmSULT expression in juvenile stages of the parasite has not yet been studied; however, OXA is known to be only slightly active against larval stages of *S. mansoni* [10]. Aiming to overcome this species and stage specificity through improved interaction with the target enzyme, we previously tested six OXA derivatives primarily based on organometallic derivatization due to its medicinal potential [11, 12] and identified three molecules (ferrocenyl, ruthenocenyl and benzyl derivatives of oxamniquine, named Fc-OXA, Rc-OXA and Bn-OXA, respectively) (Fig. 1) with a promising *in vivo* and *in vitro* activity profile against *S. mansoni.* Importantly, these compounds also displayed activity *in vitro* against *S. haematobium* [11].

The aim of the present study was to assess the damage to the tegument of *S. mansoni* and *S. haematobium*, evidenced by scanning electron microscopy (SEM) following incubation with Fc-OXA, Rc-OXA and Bn-OXA at the time points where the *in vitro* viability of the worms was markedly affected. Considering the importance of the tegument for schistosomes, which allows them to evade the immune response of the host, SEM is a valuable approach to identify whether the molecules can induce damage to this critical structure [13], and hence expose immune-reactive molecules and trigger an immune response in the host.

## Methods

### Adult *S. mansoni* worms

Three week-old female NMRI mice (*n* = 7) were purchased from Charles River and were allowed to acclimatize for one week. They were infected by a subcutaneous injection in the back of the neck with approximately 100 cercariae and kept in the animal facility of the Swiss TPH for 49 days to allow the infection to develop into the adult stage. Mice were then euthanized by the CO_2_ method and worms were collected by picking them from the hepatic portal system and mesenteric veins. Worms were washed in culture medium at room temperature and incubated at 37 °C with 5% CO_2_ until use (no longer than 2 days). The medium consisted of RPMI 1640 culture medium (Gibco - Thermofisher, Waltham, MA USA) supplemented with 1% penicillin/streptomycin (BioConcept, Allschwil, Switzerland) and 5% Fetal Calf Serum (FCS) (BioConcept). Eight to ten worms of both sexes per time point were incubated in the described medium, in the presence of 100 μM [11] of each of the three derivatives (Fc-OXA, Rc-OXA, Bn-OXA) and oxamniquine. The control consisted of 8–10 worms incubated in culture medium spiked with dimethyl sulfoxide (DMSO) at a concentration of 1%, equivalent to the content of DMSO present in the wells of treated worms. Worms were monitored daily, scoring motility, viability and morphological alterations under a bright field inverted microscope (Carl Zeiss Oberkochen, Germany, magnification ×4 and ×10) as described elsewhere [14]. The scoring scale ranges from 3 to 0 with a 0.25 interval. The score 3 corresponds to fully vital worms showing normal movement and activity, and no morphological changes; 2 is assigned to slowed worm activity, first morphological changes (loss of attachment to the well plate, suckers deformity) and visible granularity; 1 is given if minimal activity, severe morphological changes (change of the color, loss of transparency, rounded body disposition) and marked granularity is observed; and 0 refers to dead worms revealing severe granularity.

Worms were collected for SEM analysis at the time point when the average viability of the worms was visibly affected. For each compound the onset of action was different, therefore the time points range from 4 h for Fc-OXA, to 24 h for Rc-OXA, to 72 h for Bn-OXA and 120 h for OXA.

### Adult *S. haematobium* worms

One month-old male LVG hamsters (Charles River, NY) (*n* = 4) were exposed to 350 *S. haematobium* cercariae at the Biomedical Research Institute in Rockville, USA (NR-21964), and shipped to the Swiss TPH where the infection was allowed to develop for three months until chronic infections had been established. The animals were kept in the animal facility until use, for a maximum of four months. Hamsters were euthanized by the CO_2_ method and worms picked from hepatic portal system and mesenteric veins. Briefly, 8–10 adult *S. haematobium* were exposed to a 100 μM concentration for 24 h for ruthenocenyl oxamniquine, 48 h for ferrocenyl and benzyl oxamniquine and 120 h for oxamniquine, depending on the time needed for the worms to show a marked decrease in viability. Media used and scoring procedure were conducted as described for *S. mansoni* worms.

### Oxamniquine derivatives

Oxamniquine was kindly donated by Pfizer (New York City, USA) while the Fc-OXA, Rc-OXA and Bn-OXA were prepared starting from oxamniquine, as described previously [11].

### SEM images

Following the *in vitro* cultivation of the worms with the compounds as described above, *S. mansoni* and *S. haematobium* worms were rinsed twice in PBS, and fixed in 1 ml glutaraldehyde 2.5% for 4 h at room temperature. Worms were then sequentially dehydrated by incubation for 30 min in increasing concentrations of ethanol in deionized water (30, 50, 70, 90 and 100%) and kept in the fridge in 100% ethanol until use. For imaging, worms were critically point-dried (Bomar SPC-900, Washington, USA), mounted on aluminum stubs and sputter-coated with gold of 20 nm particle size (Leica EM ACE 600, Heerbrugg, Switzerland). Samples were visualized using a high-resolution SEM accelerating voltage of 5 kV (Philips XL30 ESEM, Bruchsal, Germany). Control worms were prepared and visualized in the same manner. All images were taken in the Nano Imaging Lab, SNI, University of Basel.

### IC_50_ value calculations

The IC_50_ values of each of the derivatives against *S. haematobium* were calculated using the software CompuSyn 1.0 (ComboSyn Inc, 2007) after 72 h of incubation with the following concentrations of each drug: 100, 50, 25, 12.5 and 6.25 μM. The equation to normalize the score of the treated worms to the controls and calculate the effect was:

$$ \mathrm{Effect}=1-\left(\mathrm{Average}\ {\mathrm{score}}_{\mathrm{treatment}}/{\mathrm{Average}\ \mathrm{score}}_{\mathrm{control}}\right) $$

The IC_50_ values obtained for the drugs on *S. mansoni* are described elsewhere [11].

## Results and discussion

### Studies on *S. mansoni*

**Fig. 2:**
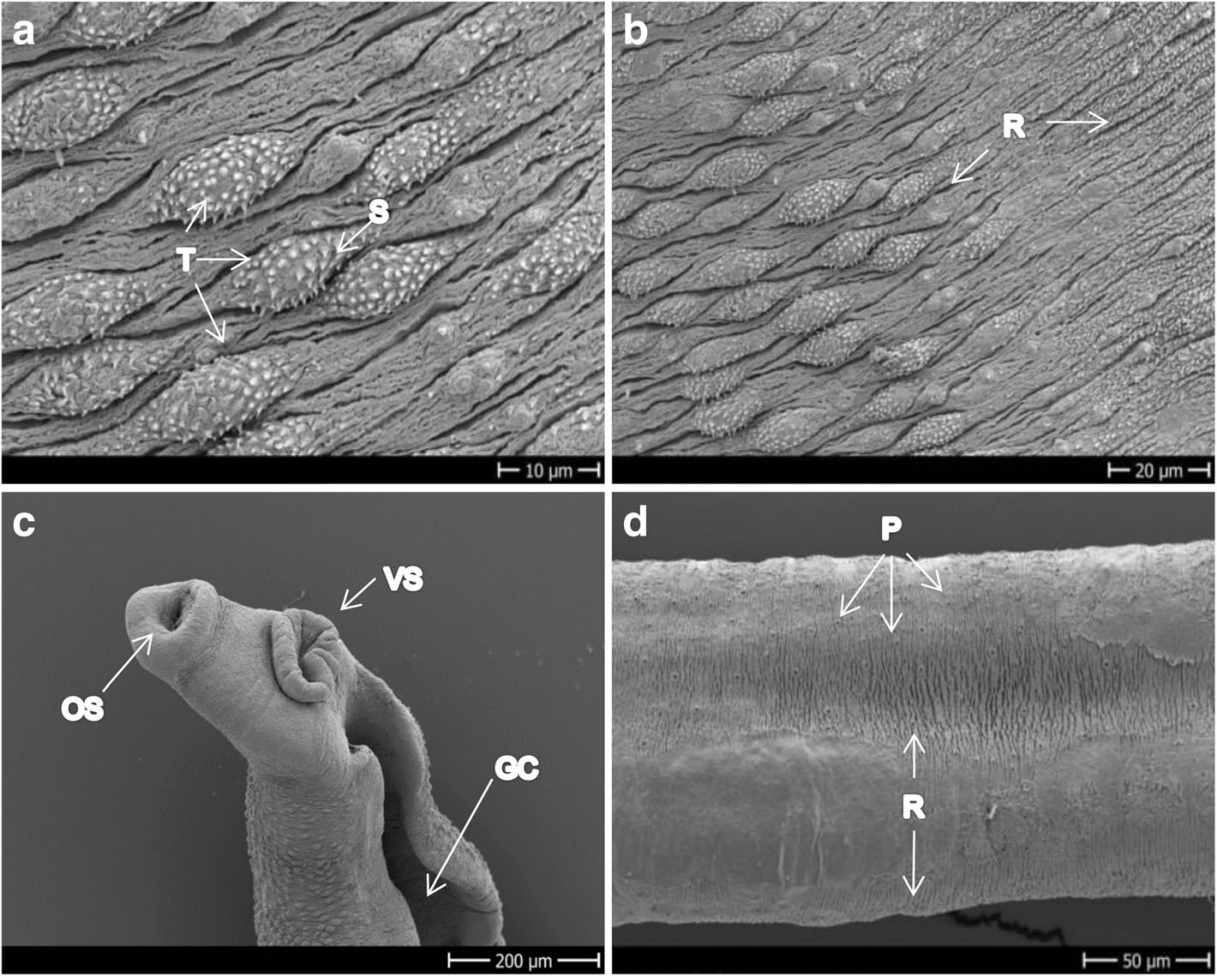
SEM images of *S. mansoni* control worms incubated with 1% DMSO. **a** Tubercles of dorsal mid-part of a male. **b** Lateral mid-part of a male showing the transition between the tubercles of the dorsal side to the spiny ridges of the ventral side of the body. **c** Head of a male showing the oral and ventral suckers and the gynaecophoric canal. **d** Mid-part of the body of a female showing the ridges and pores in a context of a smooth tegument. *Abbreviations*: GC, gynaecophoric canal; OS/VS, oral/ventral sucker; P, pores; R, ridges; S, spines; T, tubercles. *Scale-bars*: **a**, 10 μm; **b**, 20 μm; **c**, 200 μm; **d**, 50 μm

Figure 2a and b shows the tegument of healthy adult *S. mansoni* males depicting ridges and tubercles covered by spines that are more or less uniformly distributed. In Fig. 2c the oral and ventral suckers and the gynaecophoric canal of control worms are shown. Ridges are also present on females, but no tubercles or spines; instead, the teguments of the female look more porous and smooth (Fig. 2d).

After 24 h and 48 h of exposure to a 100 μM concentration of oxamniquine, the worms were alive and phenotypically in a good shape (score above 2) and we did not evidence any damage to the tegument by means of SEM (not shown). After exposure for 120 h we observed loss of attachment to the well, slight blebbing on the tegument and reduction of movement of the treated worms when compared to the control group. Since the controls also showed mild blebbing and sloughing tissue after this long incubation, we were not able to correlate a certain pattern of damage with the exposure of the worms to OXA. Our data confirm previous findings of OXA being only slightly active *in vitro* [11] and the difficulties to maintain the worms for long incubation periods where they start revealing tegument damage [15].

**Fig. 3:**
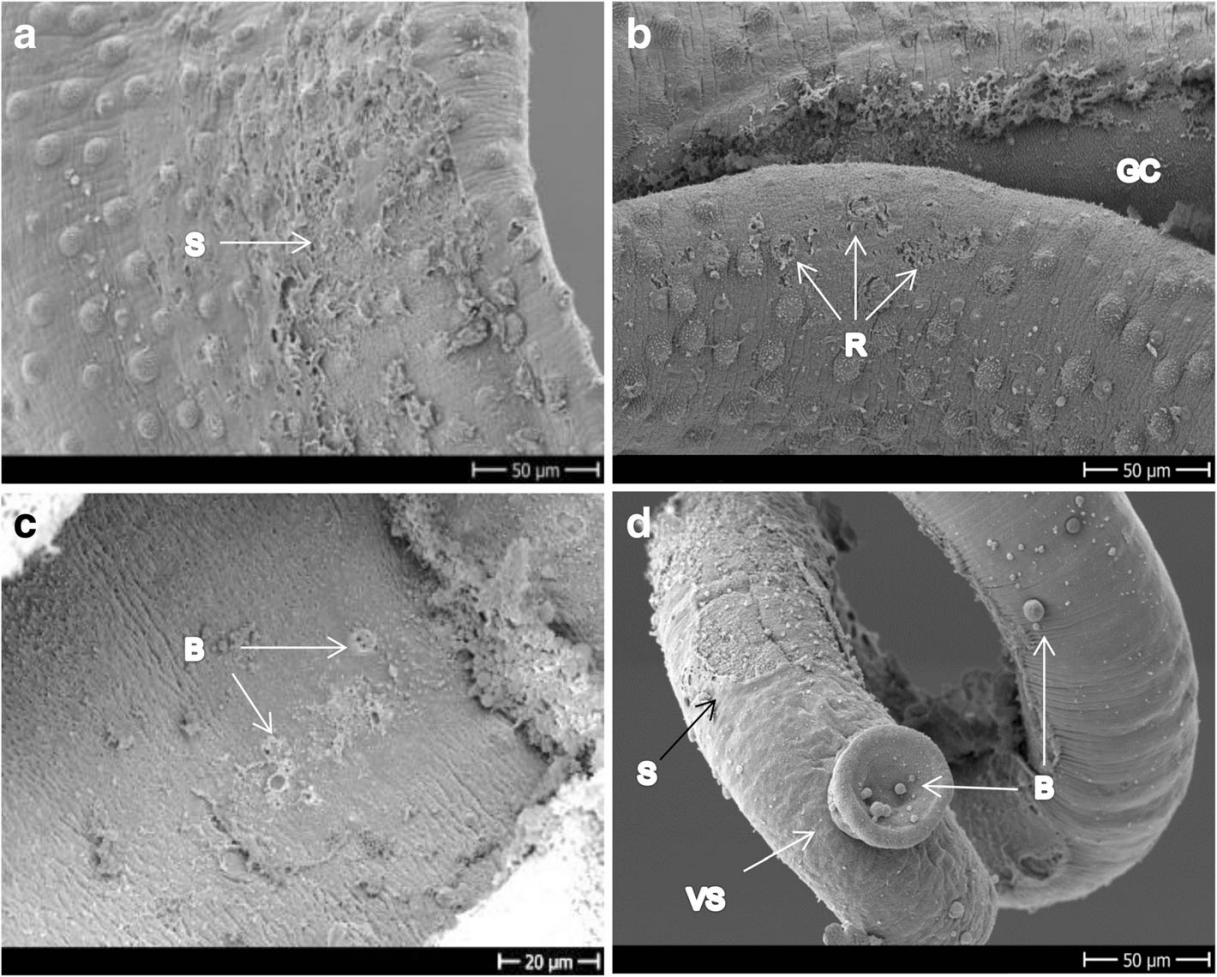
Adult *S. mansoni* exposed to 100 μM Fc-OXA incubated for 4 h. **a** Tissue sloughing from the lateral side of the mid-part of the body of a male and exposure of sub-tegumental tissue. **b** Mid-part of the body of a male showing the lateral side with ruptures, the gynaecophoric canal and ruptured tissue on the other side of the canal. **c** Blebs and ruptures in the inner side of the gynaecophoric canal, mid-part of the body. **d** Upper part of the body of a female showing tissue sloughing off and blebs on the tegument and ventral sucker. *Abbreviations*: B, blebs; GC, gynaecophoric canal; R, rupturing; S, sloughing off; VS, ventral sucker. *Scale-bars*: **a**, **b**, **d**, 50 μm; **c**, 20 μm

Fc-OXA produced after four hours of exposure at a 100 μM concentration extensive blebs, sloughing and rupture of the tegument along the whole dorsal body surface on all worms examined (Fig. 3a–c). Along the gynaecophoric canal blebbing and sloughing tissue was evident (Fig. 3c). On the males, no damage to the suckers was observed and the spines did not look significantly damaged (not shown), but were partially covered by the tissue that was detaching, as can be seen in Fig. 3a. On the females, the damage was principally focused on the upper part of the body, where Fc-OXA produced sloughing off and blebbing on the tegument and the ventral sucker (Fig. 3d).

**Fig. 4:**
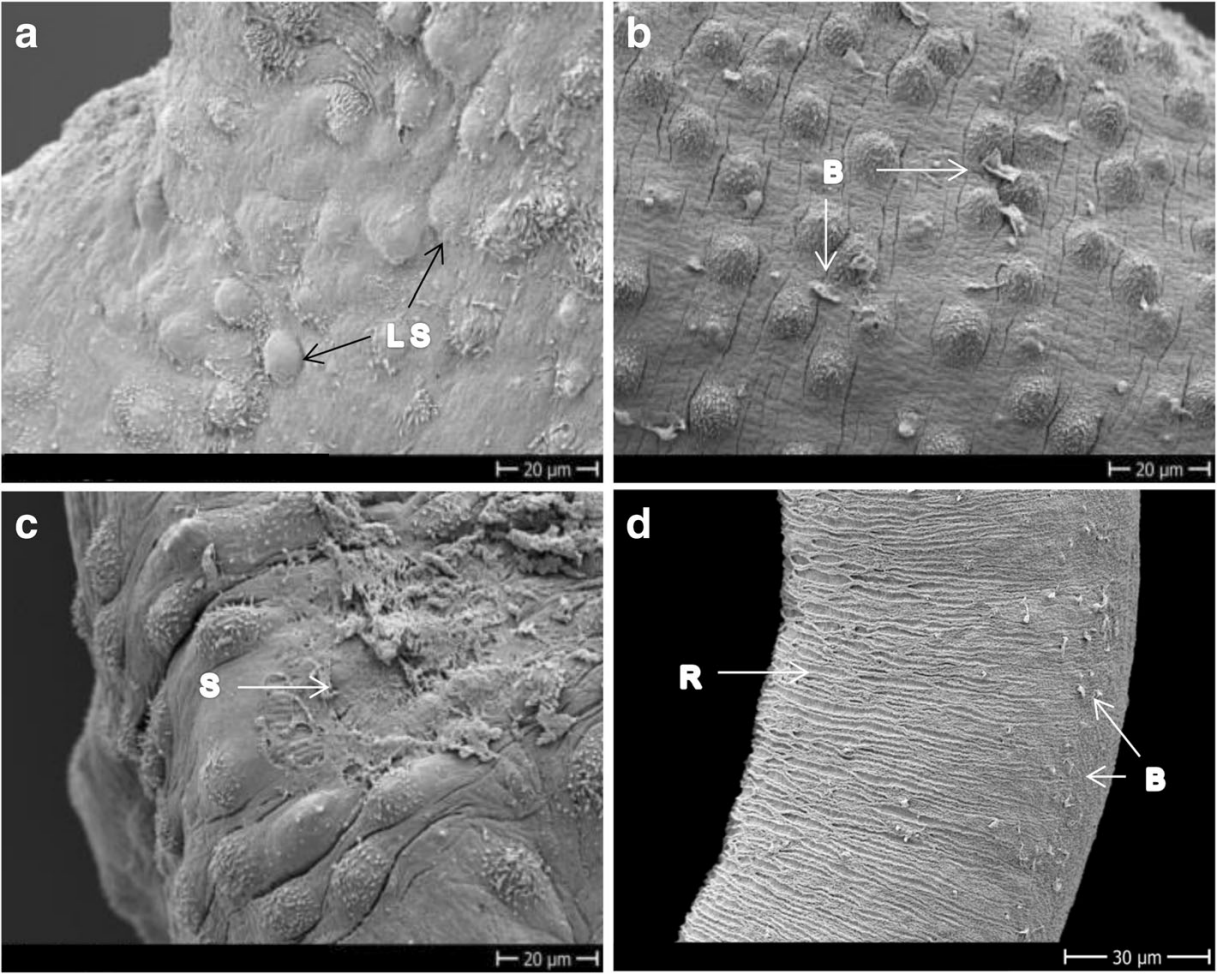
Adult *S. mansoni* exposed to 100 μM Rc-OXA for 24 h. **a**–**c** Dorsal mid-part of males showing blebs, sloughing tissue and loss of spines. **d** Mid-part of the body of a female showing undamaged ridges and very mild damage characterized by blebs along the body. *Abbreviations*: B, blebs; LS, loss of spines; R, ridges; S, sloughing off. *Scale-bars*: **a**–**c**, 20 μm; **d**, 30 μm

Four hours after exposure to Rc-OXA, the phenotypic score of the worms averaged 0.81 ± 0.15 (*n* = 8, all worms alive) yet only mild tegumental damage was observed by means of SEM, which consisted of loss of spines and mild blebs in the male, but no damage was evidenced in the females (not shown). After 24 h of incubation, the male worms had died and the females showed significant morphological changes, very low motility and the maximum score observed was 0.25. Tegumental damage of the males consisted of increased loss of spines (Fig. 4a) accompanied by tegument sloughing, blebs and vesicle formation all over the body surface (Fig. 4a–c). Although the viability score of the females was below 0.5, no pronounced tegumental damage could be evidenced using SEM (Fig. 4d). The oral and ventral suckers did not show significant damage and most couples stayed together during drug exposure (not shown) despite death of the male worms. We have no explanation for this observation, but it is possible that the females showed very low vitality and eventually were not able to dissociate from the dead male.

**Fig. 5:**
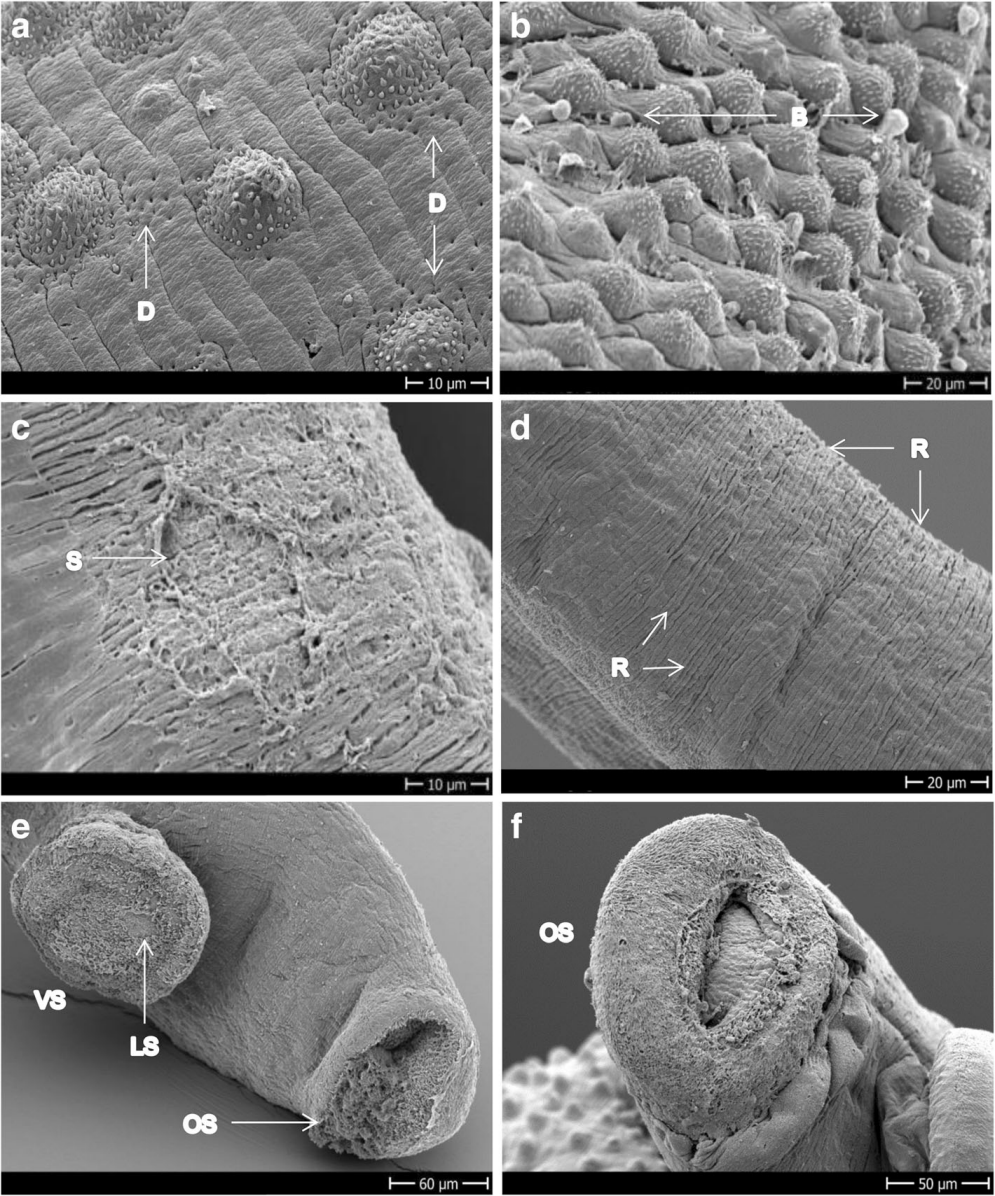
Adult *S. mansoni* exposed to 100 μM Bn-OXA. **a** Dorsal mid-part of a male exposed for 24 h showing very mild damage. **b** Dorsal mid-part of a male’s body incubated for 72 h. **c**, **d** Mid-part of female exposed for 72 h showing tegument ruptures and loss of uniformity of the ridges. **e**, **f** Head of males exposed for 72 h showing loss of spines from the ventral sucker and rupture and occlusion of the oral sucker. *Abbreviations*: B, blebs; D, dimples; LS, loss of spines; OS/VS, oral/ventral sucker; R, ridges; S, sloughing off; *Scale-bars*: **a**, **c**, 10 μm; **b**, **d**, 20 μm; **e**, 60 μm; **f**, 50 μm

Bn-OXA produced minor damage to the tegument 24 h after incubation, which consisted of small dimples (Fig. 5a). Only after 72 h of drug exposure the tegumental damage became evident and consisted, similar to Fc-OXA and Rc-OXA, of blebs (Fig. 5b), sloughing and tegumental rupture distributed all over the dorsal surface (not shown). At this time point, eight worms were dead (5 females, 3 males) while five were still alive but highly affected (scoring below 1). Of the three candidates tested, Bn-OXA was the one which showed the most visible damage to the females, which consisted of tegumental rupture (Fig. 5c) and loss of uniformity on the ridges (Fig. 5d), and surprisingly was the only compound with slightly more activity on females than on males within 72 h of drug exposure. Additionally, Bn-OXA was the only compound that produced loss of spines from the ventral sucker and erosion in the oral sucker of *S. mansoni* (Fig. 5e, f).

Table 1 summarizes IC_50_ values [11], onset of action and key tegumental changes for all compounds examined on *S. mansoni*. Bn-OXA had the slowest onset of action, which slightly contradicts the results presented by Hess et al. [11] who described 80% reduction in viability already after 24 h and 100% effect (all worms dead) in 72 h. The qualitative changes observed differ between the compounds studied and between males and females, a finding which is not uncommon for antischistosomal drugs. Artemether for example, causes extensive peeling in females but not in males [16]. Overall, the tegumental changes observed resemble findings from previous studies with praziquantel and other lead candidates, which also mention loss of spines, blebs and sloughing tissue with exposure of sub-tegumental tissue [17, 18].

**Table 1:** Comparative activity and effect of the OXA derivatives against *S. mansoni*

Compound	*S. mansoni* adult 72 h IC_50_ (μM)	Onset of action 100 μM (h)	Most affected sex	Tegumental damage		
				Blebbing	Sloughing off	Specific damage pattern/organ
Fc-OXA	11.4^a^	4	Males	+	++	Gynaecophoric canal
Rc-OXA	8.7 ^a^	24	Males	++	+	Loss of spines
Bn-OXA	11.1 ^a^	72	Females	+	+++	Suckers
OXA	> 100	120	Both	β	β	β

*Abbreviation*: β, no difference was observed to control group

^a^Hess et al. [11]

### Studies on *S. haematobium*

**Fig. 6:**
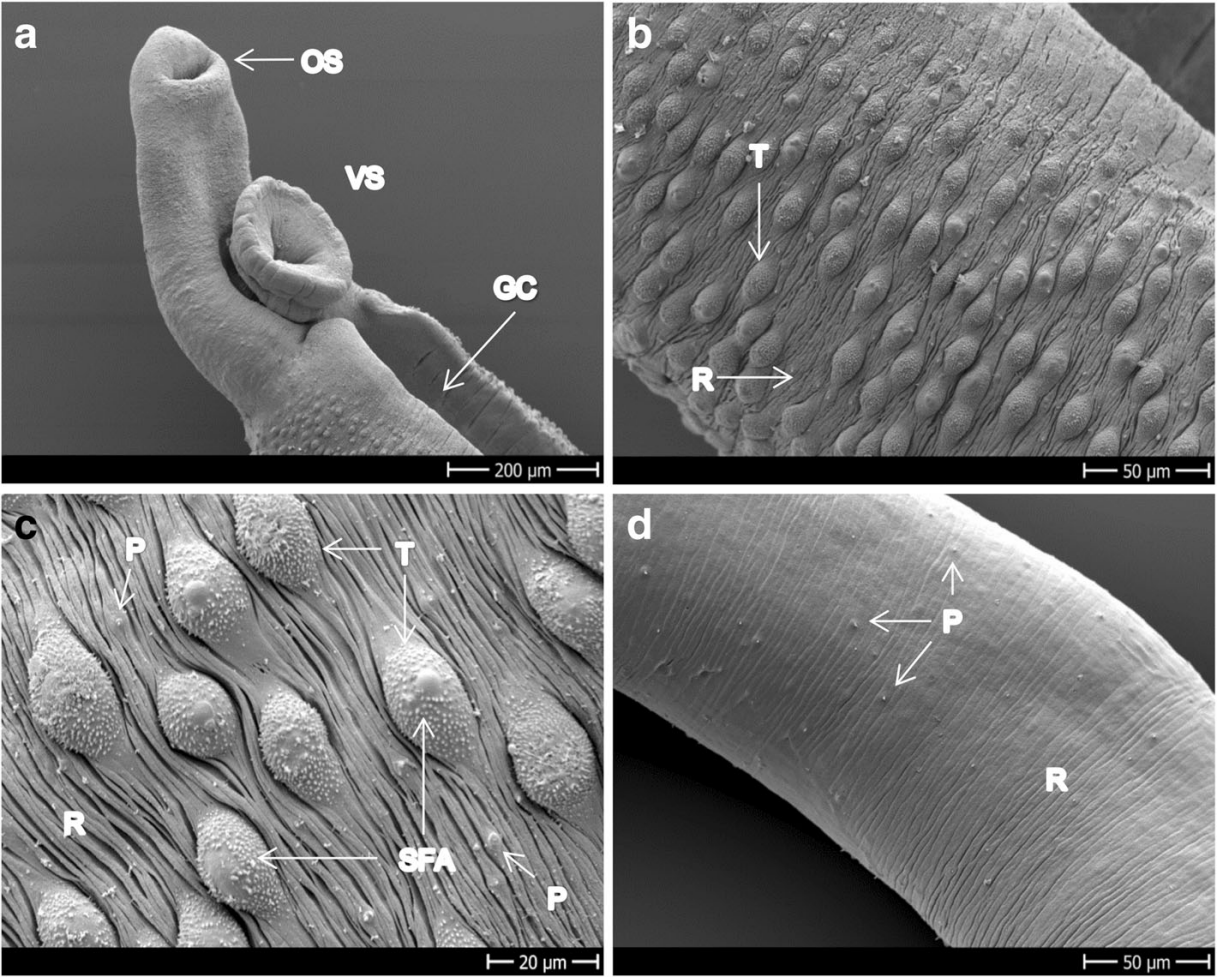
Control group of adult *S. haematobium*. **a** Head of a male showing oral and ventral suckers and the gynaecophoric canal. **b** Dorsal and lateral side of a male with tubercles and ridges. **c** Dorsal side of the mid-part of male showing ridges, sensory papillae and apex-spine-free tubercles. **d** Mid-part of the body of a female revealing the smooth tegument with ridges and sensory papillae. *Abbreviations*: GC, gynaecophoric canal; OS/VS, oral/ventral sucker; P, papillae; R, ridges; SFA, spine free apex; T, tubercles. *Scale-bars*: **a**, 200 μm; **b**, 50 μm; **c**, 20 μm; **d**, 50 μm

Figure 6 depicts images of control *S. haematobium*. Ridges and tubercles with spines on the dorsal side of the tegument and spines uniformly distributed in the gynaecophoric canal are visible (Fig. 6a, b). The apexes of the tubercles of male *S. haematobium* are free of spines (Fig. 6c). The tegument of the females also presents ridges, looks smoother than the males and the sensory papillae are uniformly distributed all over the body (Fig. 6d).

**Fig. 7:**
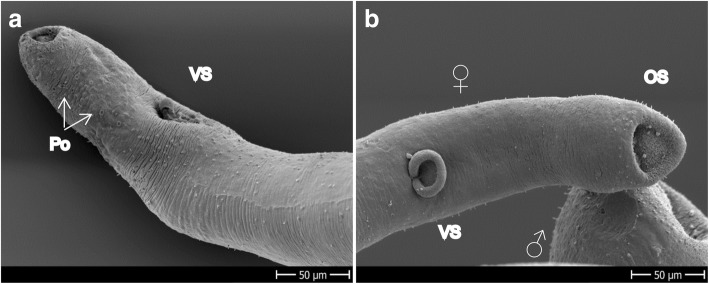
*Schistosoma haematobium* exposed to 100 μM OXA for 120 h. **a** Head of a female showing the invaginated ventral sucker. **b** Head of a female showing the invaginated ventral sucker and mild damage to the oral sucker consisting of blebs and the oral sucker of a male, also showing small ruptures and blebs. *Abbreviations*: OS, oral sucker; Po, pores; VS, ventral sucker. *Scale-bars*: 50 μm

As observed for *S. mansoni*, *S. haematobium* incubated for 120 h in 1% DMSO also showed slight tegumental damage, characterized by blebs and sloughing tissue (not shown). Therefore few conclusions can be drawn with regard to the damage of 100 μM OXA incubated for 120 h. However one sign of damage could be related to OXA, due to the fact it was not evidenced in any of the controls: worms incubated with OXA for 120 h showed invagination of the ventral sucker in females and erosion of the oral sucker in both sexes (Fig. 7a, b). Additionally, we observed a higher number of pores in both sexes of the treated worms with respect to the controls (Fig. 7a).

**Fig. 8:**
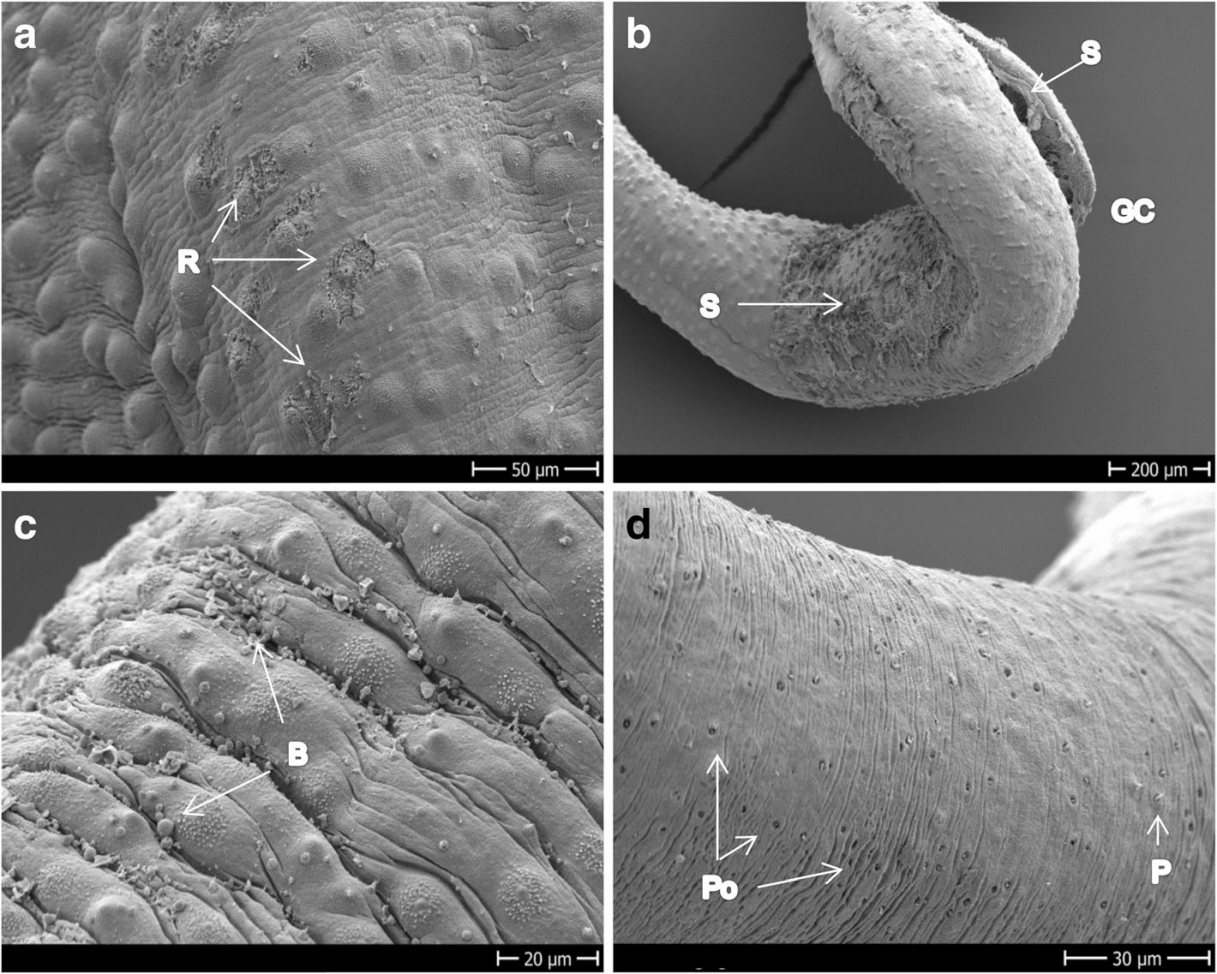
Adult *S. haematobium* exposed to 100 μM Fc-OXA for 48 h. **a** Dorsal mid-part of the body of a male showing early rupturing around the tubercles. **b** Tegument sloughing at the dorsal mid-part of the body and the gynaecophoric canal. **c** Blebs on the dorsal mid-part of the body of a male. **d** Tegument of a female showing normal ridges and papillae, but also pores; pores seem to be present in higher quantity than in the control group. *Abbreviations*: B, blebs; GC, gynaecophoric canal; P, papillae; Po, pores; R, rupturing; S, sloughing off. *Scale-bars*: **a**, 50 μm; **b**, 200 μm; **c**, 20 μm; **d**, 30 μm

After 48 h of exposure to 100 μM Fc-OXA we observed damage to the tegument and the gynaecophoric canal along the entire body, which, as in *S. mansoni*, consisted of blebbing and sloughing off (Fig. 8a–c). The sloughing tissue started its rupture from the areas surrounding the tubercles (Fig. 8a). Compared to the damage of this compound to the gynaecophoric canal in *S. mansoni*, the damage to this part of the body in *S. haematobium* was more pronounced (Fig. 8b). In females, appearance of pores over all the tegument (Fig. 8d), sporadic blebs and sloughing tissue (not shown) was visible. Damage to the oral or ventral suckers was not seen for Fc-OXA and the overall damage observed was more evident in males than in females.

**Fig. 9:**
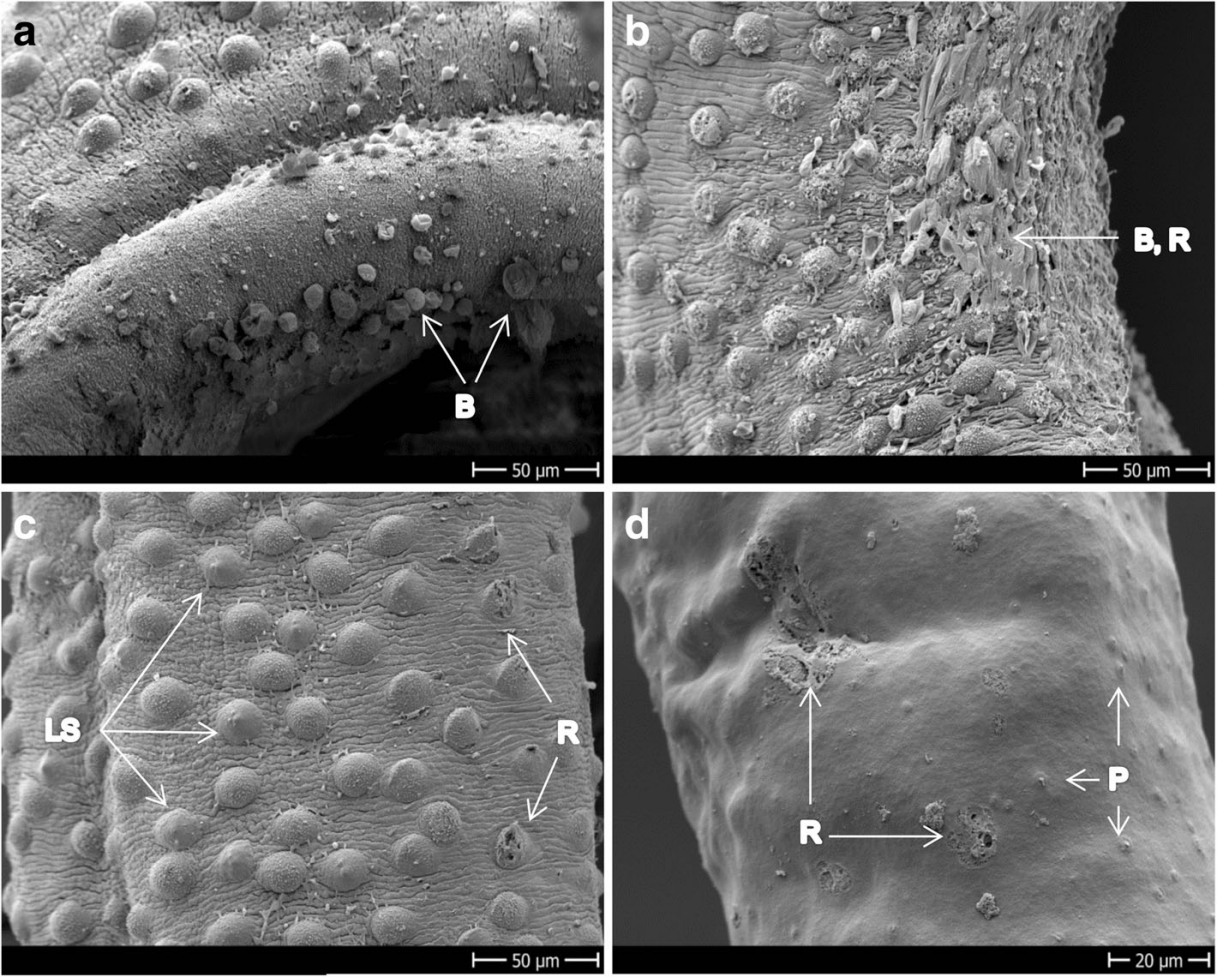
Adult *S. haematobium* exposed to 100 μM Rc-OXA for 24 h. **a**–**c** Dorsal mid part of the body of a male showing blebs, tissue rupture and loss of spines. **d** Mid-part of the body of a female showing the tegument rupture. *Abbreviations*: B, blebs; LS, loss of spines; P, papillae; R, ruptures. *Scale-bars*: **a**–**c**, 50 μm; **d**, 20 μm

The treatment of *S. haematobium* with Rc-OXA produced, as in *S. mansoni,* rupture of the tegument, with blebs and sloughing of tissue, and loss of spines within 24 h of incubation (Fig. 9a–c). In the females (Fig. 9d) we observed damage limited to small ruptures not related to any specific section or structure of the body despite that the viability of both male and female worms had decreased to a score below 0.5, revealing high activity of the drug. We could not evidence damage to the suckers or gynaecophoric canal following exposure to this compound either in males or in females.

**Fig. 10:**
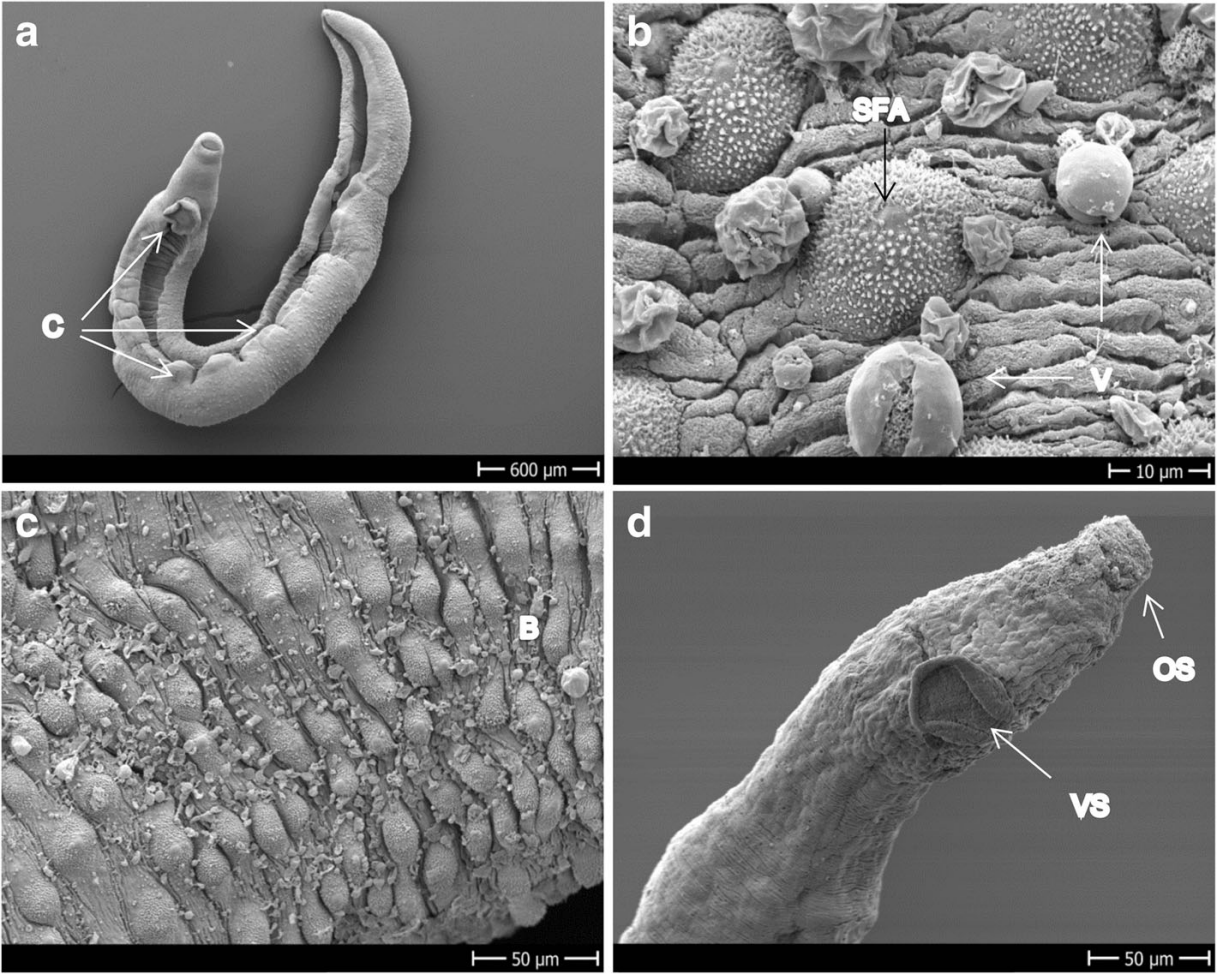
Adult *S. haematobium* exposed to 100 μM Bn-OXA for 48 h. **a** Male showing creases of the tegument and the circular disposition of the body before dying. **b**, **c** Dorsal mid-part of male’s tegument showing the extensive distribution of blebs, vesicles and mild sloughing off. **d** Head of a female showing extensive erosion of the oral sucker and extensive deformity of the ventral sucker. *Abbreviations*: B, blebs; C, creases; OS/VS, oral/ventral sucker; SFA, spine free apex; V, vesicles. *Scale-bars*: **a**, 600 μm; **b**, 10 μm; **c**, **d**, 50 μm

Following incubation of *S. haematobium* males with Bn-OXA, creases of the inner and outside of the body were visible after 48 h of exposure and there was a tendency of the worms to die in a circular body disposition (Fig. 10a). As in *S. mansoni* and Fc-OXA and Rc-OXA on *S. haematobium*, bleb/vesicles formation was a common phenomenon that appeared in large quantities on the entire surface of the tegument (Fig. 10b, c). With regard to tegumental sloughing, the effect of Bn-OXA against *S. haematobium* was much milder (Fig. 10b, c) when compared to the other compounds (Figs. 8b, 9b) and exposure of *S. mansoni* to Bn-OXA (Fig. 5c, f). We did not evidence damage to the suckers of the males following exposure to Bn-OXA*.* However, in females, the erosion and deformity to both oral and ventral suckers was significant (Fig. 10d) and was also accompanied by sloughing tissue.

Different from the activity of Bn-OXA against *S. mansoni*, in *S. haematobium* this compound was more active against males than against females, but as for *S. mansoni*, Bn-OXA seemed to reveal the highest activity of the three derivatives against females.

Table 2 summarizes *in vitro* effects of the three compounds on *S. haematobium*. After exposure to a 100 μM concentration, Fc-OXA needed 48 h to trigger a reduction in viability of 75–85% on *S. haematobium*, while Rc-OXA was lethal for all males and reduced the viability of the females to a score lower than 0.5 within 24 h. Rc-OXA also had the lowest IC_50_ value of the three compounds. Bn-OXA reduced the viability of males and females by 70% within 48 h. All derivatives were more active on males than on females, while in the case of oxamniquine no difference of sex susceptibility could be determined. Also, for *S. haematobium* we observed loss of spines and sloughing tissue, which are signs of drug activity that have been described before, for example for artemisinins against *S. haematobium* [19]. Other studies reported the damage of active compounds to *S. haematobium* to be characterized by edematous intertubercular spaces and collapse of tubercles, signs that were not observed in our compounds [20].

**Table 2:** Comparative activity and effect of the derivatives against *S. haematobium*

Compound	*S. haematobium* adult 72 h IC_50_ (μM)	Onset of action 100 μM (h)	Most affected sex	Tegumental damage		
				Blebbing	Sloughing off	Specific damage pattern/organ
Fc-OXA	68.1	48	Males	+	++	Gynaecophoric canal
Rc-OXA	24.5	24	Males	++	++	Loss of spines
Bn-OXA	38.6	48	Males	+	+++	Body creases
OXA	> 100	120	Both	+	β	VS invagination

*Abbreviations*: VS, ventral sucker; β, no difference was observed to control group

### Comparison of oxamniquine derivatives

While the fastest and most active compound against *S. mansoni* was Fc-OXA, Rc-OXA was the most promising compound for *S. haematobium* with regard to *in vitro* activity and tegumental damage. Surprisingly, Fc-OXA had the highest IC_50_ value against *S. haematobium.* Bn-OXA was the compound with the slowest visible effect against both species, but was active against both species as well, in a timeframe of 48–72 h, depending on the species. Overall, a higher activity of the derivatives was observed against *S. mansoni* than against *S. haematobium*.

For both species, and all three derivatives investigated, we could observe common patterns of damage to the tegument, which consisted of extensive blebs and considerable tegument rupture and sloughing off along the whole body, with exposure of sub-tegumental tissue. The sub-tegument might expose antigens and therefore trigger an immune response attack, commonly observed for antischistosomal drugs [17–19].

Differences in the damage distribution cannot be explained, but might be due to a differential distribution of the activating enzyme in the body of the parasites or additional mechanisms of action for the individual derivatives supporting the antiparasitic effect.

## Conclusions

Our study confirmed that Fc-OXA, Rc-OXA and Bn-OXA are promising broad spectrum antischistosomal drug candidates. All derivatives showed high *in vitro* activity against *S. mansoni* and *S. haematobium*, while validating the previous finding that the parent drug oxamniquine is less active *in vitro* under the conditions described. Future studies should aim to further characterize these compounds, taking into account the *in vivo* activity on juvenile stages of development and activity against *S. haematobium in vivo*.

## Abbreviations

OXA: Oxamniquine
Fc-OXA: Ferrocenyl oxamniquine
Rc-OXA: Ruthenocenyl oxamniquine
Bn-OXA: Benzyl oxamniquine
SEM: Scanning electron microscopy
SmSULT: *Schistosoma mansoni* sulfotransferase
DMSO: Dimethyl sulfoxide
